# Influence of Placement Techniques on Marginal Integrity, Wear Behavior, and Clinical Efficiency of a Bulk-Fill Resin Composite

**DOI:** 10.3390/jfb17030108

**Published:** 2026-02-24

**Authors:** Kerem Can Işık, Handan Yıldırım-Işık, Uğur Tuna Sazlıkoğlu, Mediha Büyükgöze-Dindar

**Affiliations:** 1 Department of Restorative Dentistry, Faculty of Dentistry, Istanbul Beykent University, Istanbul 34396, Turkey; kerem.isik@beykent.edu.tr (K.C.I.); handanyildirim@beykent.edu.tr (H.Y.-I.); 2 Department of Prosthodontics, Faculty of Dentistry, Istanbul Beykent University, Istanbul 34396, Turkey; tunasazlikoglu@beykent.edu.tr; 3 Department of Restorative Dentistry, Faculty of Dentistry, Trakya University, Edirne 22030, Turkey

**Keywords:** composite resins, dental leakage, dental restoration wear, operative time

## Abstract

The placement technique of resin composites may significantly influence marginal integrity, wear resistance, and operative efficiency. This in vitro study evaluated the influence of different placement techniques for a bulk-fill resin composite on marginal integrity, wear behavior, and application time. Standardized Class I cavities were prepared in extracted human molars and restored using the same bulk-fill composite (Filtek One Bulk Fill, 3M, USA) applied with four techniques: incremental placement, incremental placement with a modeling liquid (GC Modeling Liquid, GC Corp., Tokyo, Japan), bulk placement, and the stamp technique. Application time was recorded in seconds. All specimens underwent combined mechanical and thermal aging (SD Mechatronik, Germany). Marginal integrity was assessed three-dimensionally using micro-computed tomography, while surface wear was quantified through computer-based digital analysis with OraCheck software (Dentsply Sirona, Germany). Bulk placement exhibited significantly higher microleakage scores than the other techniques while demonstrating the shortest application time. Incremental placement, incremental placement with modeling liquid, and the stamp technique showed comparable microleakage results (*p* > 0.05). Although the use of modeling liquid did not increase microleakage, it resulted in significantly greater wear. Placement technique significantly influences marginal integrity, wear behavior, and application time of bulk-fill composite restorations.

## 1. Introduction

Direct resin-based composites are widely used in contemporary restorative dentistry owing to their ability to restore lost tooth structure while maintaining acceptable mechanical performance and aesthetic integration. Ongoing refinements in composite formulation—including advances in filler architecture, resin chemistry, and light-activation systems—have improved their durability and expanded their applicability in posterior regions exposed to high occlusal stresses [1]. In addition, the minimally invasive nature of direct composite restorations, together with reduced treatment cost and chairside time, has contributed to their widespread clinical adoption [2]. The moldable characteristics of composite resins further allow precise reproduction of tooth anatomy, supporting their use in conservative and efficient restorative procedures [3].

Nevertheless, polymerization-related volumetric contraction remains an unavoidable characteristic of resin composite materials [4]. The stresses generated during the curing process may disrupt the adhesive interface between the restoration and the surrounding tooth tissues, particularly in cavities with a high ratio of bonded surfaces. Such stress concentration can result in interfacial gaps that facilitate microleakage, defined as the subclinical ingress of fluids, microorganisms, or dissolved substances along the tooth–restoration interface [5]. Microleakage has been implicated in several undesirable clinical outcomes, including marginal staining, postoperative sensitivity, pulpal irritation, and the development of secondary caries, all of which may compromise restoration longevity [6].

The extent to which polymerization stress affects marginal integrity is strongly influenced by cavity configuration. Class I cavities, characterized by a high configuration factor (C-factor), are especially vulnerable to shrinkage-induced stress accumulation [7]. To mitigate these effects, incremental placement techniques have traditionally been recommended. In this approach, resin composites are placed in increments of up to 2 mm, each cured separately, allowing adequate light penetration and monomer conversion [8]. However, incremental techniques are technique-sensitive, increase chairside time, and may be associated with void formation, interlayer contamination, or bonding failures, especially in large cavities [9]. Additionally, the complexity of the protocol may compromise clinical efficiency, despite the potential for improved anatomical and aesthetic control.

In response to these challenges, bulk-fill resin composites have been developed to allow placement in thicker layers while maintaining sufficient depth of cure and controlled polymerization stress [10]. Modifications in optical properties and polymerization kinetics permit single-increment placement, offering a simplified restorative approach. Alongside material innovations, alternative application methods—such as the stamp technique or surface refinement using brush application in combination with a modeling liquid—have been proposed to improve anatomical reproduction, marginal adaptation, and surface quality [3,11]. However, despite their increasing clinical use, the influence of these placement techniques on microleakage and long-term surface behavior remains controversial.

Beyond interfacial integrity, the long-term success of resin composite restorations is also closely related to their mechanical stability and resistance to functional wear. In the oral environment, restorative materials are exposed to complex tribological conditions involving cyclic occlusal loading, sliding contact, and abrasive interactions with opposing dentition and food particles. Studies in polymer composite science have consistently shown that wear behavior is governed by filler characteristics, reinforcement distribution, interfacial adhesion, applied load, and environmental conditions [12]. Improved filler–matrix coupling and optimized micro–nano reinforcement strategies have been associated with enhanced hardness, reduced friction coefficient, and superior resistance to abrasive and fatigue wear. Consequently, any clinical variable that alters surface morphology or structural continuity—such as placement technique—may potentially influence not only marginal integrity but also mechanical and tribological performance over time.

Current literature provides limited and sometimes inconsistent data regarding the combined effects of placement technique, surface characteristics, and aging on the performance of bulk-fill composite restorations [8,13]. Therefore, the objective of this in vitro study was to assess the microleakage behavior of a contemporary bulk-fill resin composite applied using four different placement techniques—bulk placement, stamp technique, incremental placement, and incremental placement combined with brush application using a modeling liquid—in standardized Class I cavities. In addition, restorative procedure time, marginal adaptation, surface characteristics, and wear behavior were evaluated following artificial aging. The null hypothesis was that the placement technique would not significantly influence microleakage, surface properties, or procedural efficiency of bulk-fill composite restorations.

## 2. Materials and Methods

### 2.1. Ethical Approval and Funding

This in vitro investigation was conducted following approval from the Ethics Committee of Istanbul Beykent University Faculty of Dentistry [approval date: 28 February 2024; Decision No: 2024/2]. Financial support for the study was provided by the Beykent University Scientific Research Projects Coordination Unit [Project No: 2023-24-BAP-07, 2024].

### 2.2. Sample Size Determination

The sample size was calculated based on previously published data [14], assuming an effect size of 0.63, a significance level (α) of 0.05, and a statistical power of 95%. According to these parameters, a minimum of 12 specimens per group was required, resulting in a total sample size of 48 teeth.

### 2.3. Specimen Selection and Storage

Forty-eight extracted human permanent molars, including maxillary and mandibular teeth, were included in the study. Teeth extracted for orthodontic or periodontal reasons were eligible. Third molars were also considered; however, those presenting complex occlusal morphology were excluded to ensure cavity standardization and minimize anatomical variability. Only teeth free from caries, restorations, cracks, fractures, or other structural defects were included.

Following extraction, residual soft tissues were mechanically removed, and the specimens were stored in distilled water at 4 °C for a maximum of four weeks prior to cavity preparation to preserve dentinal structure and hydration.

### 2.4. Cavity Preparation

**Figure 1:**
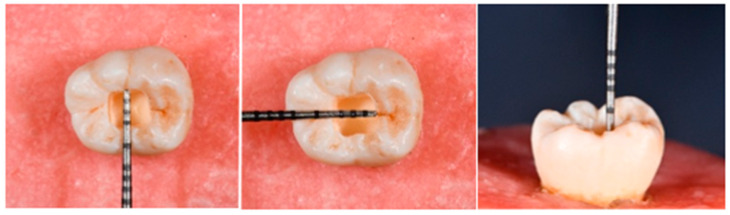
Cavity preparation.

Standardized Class I cavities were prepared in the central region of the occlusal surface of each tooth using a water-cooled high-speed handpiece and a cylindrical medium-grit diamond bur (100 μm; 842, Komet, Lemgo, Germany). The cavities were designed with a box-shaped configuration measuring 3 mm × 3 mm in width and 4 mm in depth, with the pulpal floor located at the level of mid-coronal dentin (Figure 1). All preparations were examined under magnification to verify the absence of enamel–dentin cracks and to exclude any specimens with pulpal exposure. To ensure consistency, the diamond bur was replaced after every five cavity preparations.

Following cavity preparation, the specimens were randomly allocated into four experimental groups (n = 12) according to the placement technique employed:

Group 1: Bulk-fill placement technique

Group 2: Stamp technique

Group 3: Incremental placement technique

Group 4: Incremental placement combined with brush application using a modeling liquid

Detailed information regarding the materials employed, including their compositions, is provided in Table 1. All cavities were restored using the same bulk-fill resin composite material (Filtek One Bulk Fill, 3M, St. Paul, MN, USA). Adhesive procedures were performed using a two-step self-etch adhesive system (CLEARFIL SE BOND; Kuraray Noritake Dental, Tokyo, Japan), applied strictly in accordance with the manufacturer’s instructions. In Group 4, following incremental placement of the composite resin, the surface of each increment was gently refined using a disposable microbrush lightly moistened with a modeling liquid (GC Modeling Liquid, GC Corporation, Tokyo, Japan) prior to light curing. This procedure was performed to facilitate surface smoothing and anatomical contouring while minimizing surface irregularities.

Light activation of both the adhesive and restorative material was carried out using a light-emitting diode (LED) curing unit (Woodpecker iLed II; Woodpecker Medical Instrument Co., China) operating at an output intensity of approximately 1000–1200 mW/cm^2^, as specified by the manufacturer. The same curing unit was used for all specimens, and light output was periodically verified to ensure consistent irradiance throughout the experimental procedures.

To minimize procedural variability, a single adhesive system and identical light-curing protocol were applied to all groups. Furthermore, all restorative procedures were performed by a single experienced operator, thereby reducing operator-related bias and enhancing methodological consistency.

### 2.5. Procedure Time Measurement

Procedure time was recorded for each restoration using a digital stopwatch. Timing commenced at the initiation of composite placement following completion of the adhesive procedure and concluded upon completion of the final light-curing step. The measured duration encompassed all procedural steps directly associated with composite application, anatomical shaping, and polymerization, while excluding cavity preparation, adhesive application, and any finishing or polishing procedures.

For the stamp technique group, the time required for fabrication of the stamp index was recorded separately but was not included in the primary procedure time analysis. As stamp fabrication was performed prior to adhesive application and isolation, it did not prolong the composite placement phase. To maintain methodological standardization and ensure valid comparison among placement techniques, the primary analysis was restricted to the active restorative phase.

### 2.6. Artificial Aging Procedure

**Figure 2:**
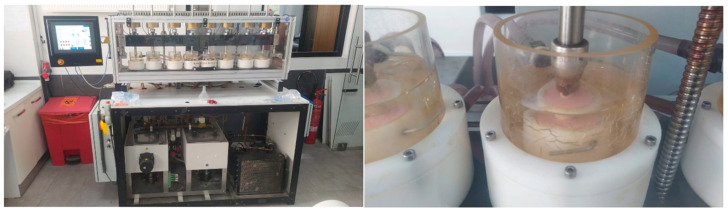
Artificial Aging Procedure.

All specimens were embedded in autopolymerizing acrylic resin blocks up to approximately 2 mm apical to the cemento-enamel junction to simulate periodontal support and ensure stable positioning during cyclic loading. To simulate long-term intraoral conditions and reproduce functional masticatory stresses, all restored specimens were subjected to combined mechanical and thermal aging using a dual-axis chewing simulator (CS-4, SD Mechatronik GmbH, Feldkirchen-Westerham, Germany) (Figure 2).

Mechanical loading was applied using a stainless-steel spherical antagonist (diameter: 4 mm), providing dynamic two-body contact with a sliding component across the occlusal surface. A vertical load of 49 N was applied at a frequency of 1.6 Hz for a total of 1,200,000 cycles, corresponding to approximately five years of simulated clinical function [15,16].

The vertical (Z-axis) stroke was set at 2.0 mm with a movement speed of 60 mm/s, while the horizontal (X-axis) stroke was adjusted to 0.5 mm with a sliding speed of 60 mm/s in a reciprocating motion [17]. This loading configuration generated controlled sliding contact over the restoration surface rather than static point loading, thereby promoting clinically relevant wear patterns.

Simultaneously, integrated thermal cycling was performed between 5 °C and 55 °C to simulate intraoral temperature fluctuations associated with hot and cold intake. Each thermal bath included a dwell time of 30 s with automated transfer between temperature chambers, ensuring standardized thermal exposure throughout the aging protocol.

The selected loading parameters, including force, frequency, stroke lengths, and movement speeds, were adopted in accordance with previously published chewing simulation protocols to ensure methodological consistency and clinical relevance [15,16,17].

The combined mechanical and thermal aging protocol was designed to provide a more clinically relevant simulation of intraoral conditions compared to static aging methods.

### 2.7. Wear Resistance Evaluation

Wear was assessed using three-dimensional (3D) deviation analysis based on superimposition of pre-aging and post-aging digital surface scans. Digital impressions of each restoration were obtained using a computer-aided design (CAD) system (Dentsply Sirona Deutschland GmbH, Bensheim, Germany) prior to artificial aging and repeated after completion of the aging protocol.

The baseline and post-aging datasets were imported into OraCheck Software (Version 5.0.x, Dentsply Sirona Deutschland GmbH, Bensheim, Germany) and registered using a best-fit alignment algorithm. Superimposition was performed based on stable, non-restored reference areas to ensure accurate spatial correspondence between the two datasets and to minimize alignment-related discrepancies.

Following superimposition, quantitative assessment of surface material loss was conducted by calculating vertical surface deviations between the two datasets. Wear was defined as vertical material loss and expressed in millimeters (mm). Negative deviation values represented loss of restorative material, whereas positive values indicated minor discrepancies attributable to alignment or measurement variability.

For standardization, the prepared Class I cavities had a fixed mesiodistal dimension of 3 mm. Measurement points were digitally defined within the restoration boundaries according to this known dimension. The mesial and distal reference points were positioned 0.75 mm inward from the respective cavity margins, and the central reference point was located at the geometric center of the restoration. This protocol ensured a consistent inter-point distance of 0.75 mm between adjacent measurement sites. The mean of the three vertical deviation values was calculated and recorded as the wear value for each specimen.

### 2.8. Microleakage Assessment

Following artificial aging, all specimens were immersed in a 50% silver nitrate (AgNO_3_) solution for 24 h under dark conditions. Subsequently, three-dimensional images were acquired using a micro-computed tomography system (Bruker SkyScan 1272, Coventry, UK).

Reconstructed horizontal and vertical cross-sectional images were systematically evaluated under standardized magnification and calibration settings. All sections of each specimen were reviewed to ensure comprehensive assessment. To maintain methodological consistency and prevent underestimation of interfacial leakage, the cross-sectional slice exhibiting the greatest extent of dye penetration was selected for quantitative analysis.

Microleakage was assessed on the reconstructed images using an ordinal scoring system based on the depth of tracer penetration relative to cavity depth, as follows:

Score 0: No detectable microleakage

Score 1: Penetration involving up to one-third of the cavity depth

Score 2: Penetration involving one-third to two-thirds of the cavity depth

Score 3: Penetration extending throughout the entire cavity depth

Quantitative linear measurements of dye penetration were performed using ImageJ software (version 1.53t, National Institutes of Health, Bethesda, MD, USA) by a single blinded operator to ensure methodological standardization and minimize measurement bias. Prior to analysis, the software was calibrated with a standardized reference distance. All measurements were conducted at 400× magnification under uniform evaluation parameters to ensure reproducibility and consistency.

In addition to quantitative microleakage scoring, qualitative interfacial evaluation was performed using scanning electron microscopy (SEM) to assess marginal characteristics and surface morphology. Representative micrographs were obtained under standardized imaging conditions to enable descriptive comparison among groups. Qualitative observations were used to support and contextualize the quantitative findings.

### 2.9. Statistical Analysis

Statistical analyses were conducted using SPSS statistical software (version 23.0; IBM Corp., Chicago, IL, USA). Data distribution was evaluated through examination of skewness and kurtosis indices, supplemented by the Shapiro–Wilk test for assessment of normality. For variables demonstrating normal distribution, intergroup comparisons were performed using one-way analysis of variance (ANOVA), followed by Tukey’s honestly significant difference test for multiple comparisons. When the assumptions of parametric testing were not fulfilled, nonparametric statistical procedures were employed, including the Kruskal–Wallis test with subsequent pairwise comparisons conducted using the Mann–Whitney U test. Associations among application time, surface wear, and microleakage parameters were investigated using correlation analysis. All quantitative data were expressed as mean ± standard deviation, and the level of statistical significance was set at *p* < 0.05.

## 3. Results

**Figure 3:**
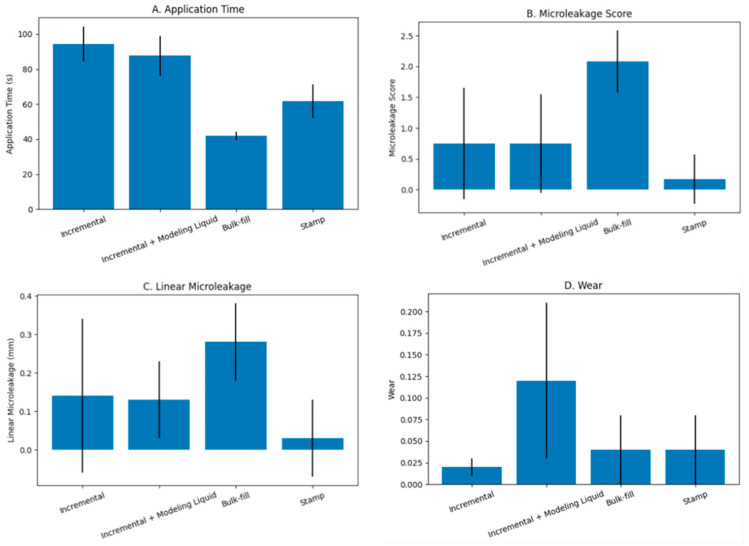
Comparison of (**A**) application time, (**B**) microleakage score, (**C**) linear microleakage, and (**D**) wear among the different composite placement techniques.

Descriptive statistics for application time, microleakage score, linear microleakage depth, and wear values according to the restorative technique are summarized in Table 2. Statistically significant differences were observed among the experimental groups for all evaluated parameters (*p* < 0.05), (Figure 3).

Intra-examiner reliability was evaluated using the intraclass correlation coefficient (ICC) for linear microleakage measurements, demonstrating excellent reliability. The ICC for single measurements was 0.970 (95% CI: 0.947–0.983), while the ICC for average measurements was 0.985 (95% CI: 0.973–0.991) (*p* < 0.001). These results indicate a high level of measurement consistency and reproducibility.

### 3.1. Procedure Time

Application time differed significantly among the placement techniques (*p* < 0.001). The bulk technique demonstrated the shortest application time (41.8 ± 2.6 s), which was significantly lower than that of all other groups. The stamp technique required a longer application time (61.7 ± 9.5 s) than the bulk technique but remained significantly shorter than both incremental techniques. No statistically significant difference was detected between the incremental technique (94.3 ± 9.9 s) and the incremental technique combined with brush application using modeling liquid (87.6 ± 11.5 s).

The separately recorded stamp index fabrication time was 15.2 ± 2.1 s. When this additional step is included, the total procedure time of the stamp technique remains shorter than that of both incremental techniques but longer than that of the bulk technique.

### 3.2. Microleakage Score

**Figure 4:**
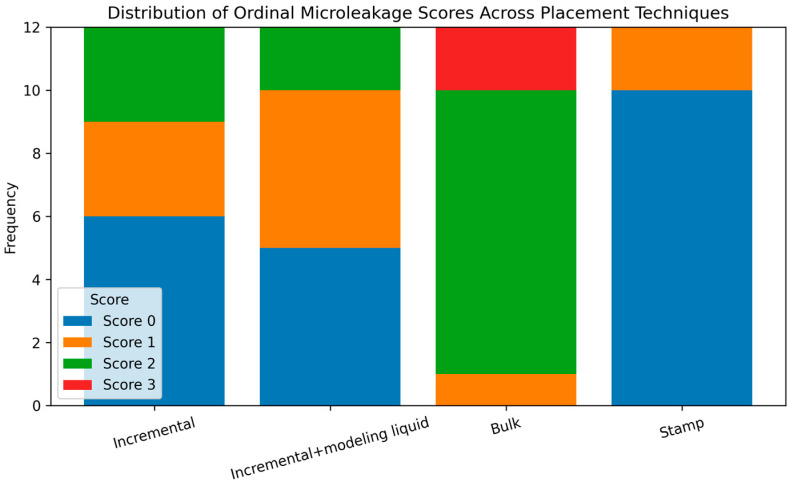
Stacked bar chart illustrating the distribution of ordinal microleakage scores (0–3) across the experimental placement techniques (incremental technique, incremental technique with modeling liquid, bulk technique, and stamp technique). The figure presents the proportional distribution of scores within each group, enabling visual comparison of leakage severity patterns among techniques.

Significant differences were found among the groups with respect to microleakage scores (*p* < 0.001) (Table 3). The bulk technique demonstrated the highest median microleakage score [2 (1–3)], which was significantly greater than those of the other groups. In contrast, the incremental technique [0.5 (0–2)], incremental technique combined with brush application using modeling liquid [1 (0–2)], and the stamp technique [0 (0–1)] exhibited lower and statistically comparable microleakage scores, with no significant differences among these groups (Figure 4). These findings indicate that bulk placement was associated with increased interfacial leakage compared with the alternative placement strategies.

### 3.3. Microleakage Measurement

**Figure 5:**
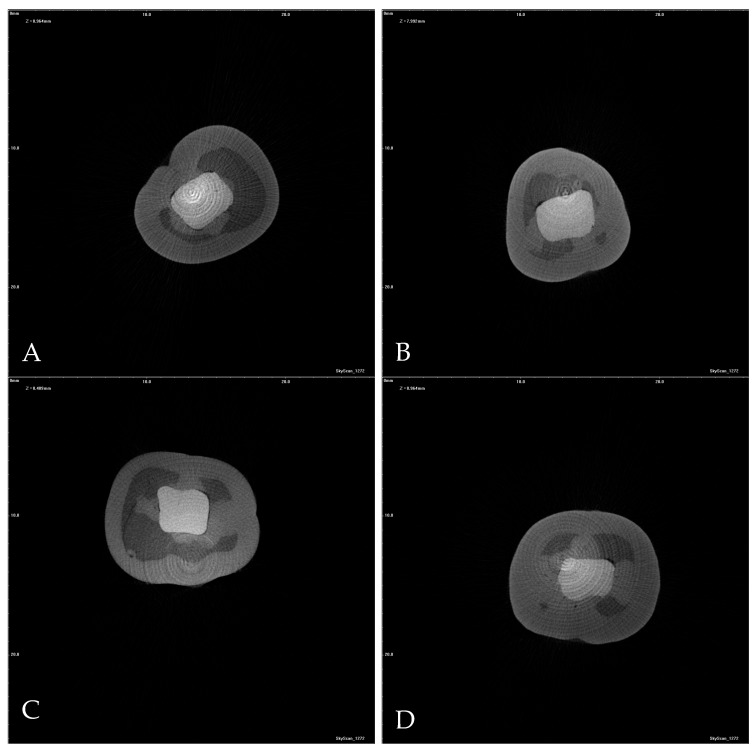
Representative micro-computed tomography cross-sectional images of the restored specimens after artificial aging. For each specimen, the section exhibiting the maximum extent of microleakage was selected for analysis. (**A**) Bulk placement technique; (**B**) Incremental placement combined with brush application using a modeling liquid; (**C**) Conventional incremental placement; (**D**) Stamp technique.

Linear microleakage values measured in millimeters showed statistically significant differences among the experimental groups (*p* = 0.001). The greatest depth of dye penetration was observed in the bulk technique group (0.28 ± 0.1 mm), which was significantly higher than that recorded for the remaining techniques. No statistically significant differences were detected among the incremental technique (0.14 ± 0.2 mm), incremental technique combined with brush application using modeling liquid (0.13 ± 0.1 mm), and stamp technique (0.03 ± 0.1 mm) (Figure 5).

### 3.4. Wear

**Figure 6:**
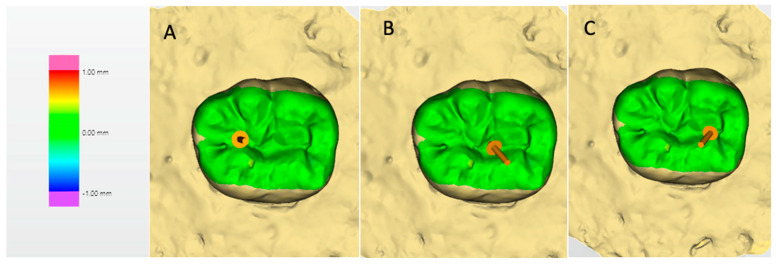
Representative images of wear evaluation using three-dimensional (3D) deviation analysis obtained by superimposition of pre- and post-aging scans (OraCheck Software, Dentsply Sirona, Germany). (**A**) Mesial reference point used for vertical wear measurement; (**B**) Central reference point; (**C**) Distal reference point. Wear was defined as the mean vertical material loss measured at three standardized reference points and is expressed in millimeters (mm). In the color-coded maps, negative values indicate material loss, whereas positive values represent surface gain or minor alignment-related deviations.

Wear values differed significantly among the placement techniques (*p* < 0.001) (Figure 6). The incremental technique combined with brush application using modeling liquid exhibited the highest mean wear value (0.12 ± 0.09 mm), and this value was significantly greater than those observed in all other groups (*p* < 0.05).

The bulk placement technique (0.04 ± 0.04 mm) and the stamp technique (0.04 ± 0.04 mm) demonstrated comparable wear values, with no statistically significant difference between them (*p* > 0.05). The conventional incremental technique exhibited the lowest wear value (0.02 ± 0.01 mm), which was significantly lower than that of the incremental technique combined with modeling liquid application (*p* < 0.05).

Overall, the incorporation of modeling liquid during incremental placement was associated with a marked increase in surface wear compared with the other placement techniques.

### 3.5. SEM Analysis

**Figure 7:**
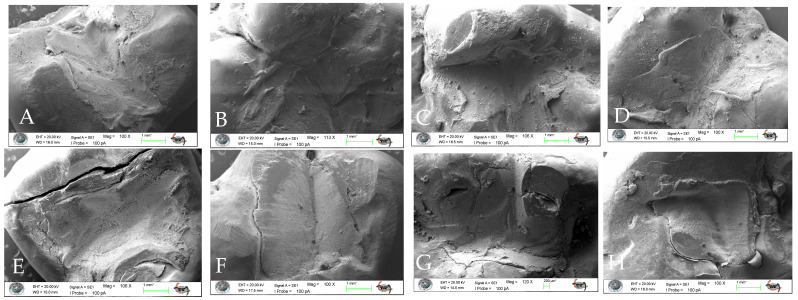
Scanning electron microscopy (SEM) images of the specimens at 100× magnification. (**A**): Bulk technique—baseline; (**E**): Bulk technique—after aging; (**B**): Incremental technique combined with brush application using a modeling liquid—baseline; (**F**): Incremental technique combined with brush application using a modeling liquid—after aging; (**C**): Incremental technique—baseline; (**G**): Incremental technique—after aging; (**D**): Stamp technique—baseline; (**H**): Stamp technique—after aging.

**Figure 8:**
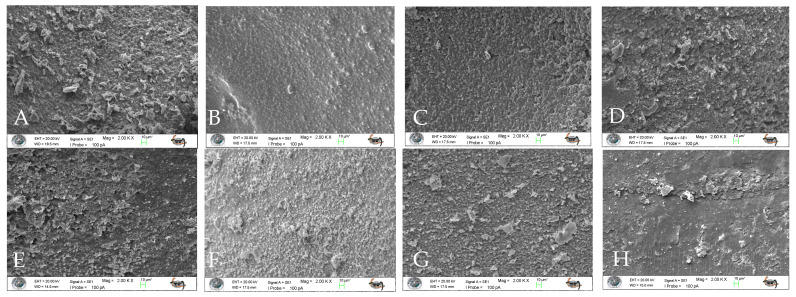
Scanning electron microscopy (SEM) images of the specimens at 2000× magnification. (**A**): Bulk technique—baseline; (**E**): Bulk technique—after aging; (**B**): Incremental technique combined with brush application using a modeling liquid—baseline; (**F**): Incremental technique combined with brush application using a modeling liquid—after aging; (**C**): Incremental technique—baseline; (**G**): Incremental technique—after aging; (**D**): Stamp technique—baseline; (**H**): Stamp technique—after aging.

Surface characteristics and marginal adaptation of the specimens were assessed using scanning electron microscopy (SEM) at different magnifications. Representative SEM micrographs obtained at 100× and 2000× magnifications are presented in Figure 7 and Figure 8, respectively.

SEM analysis revealed that aging performed with the chewing simulator adversely affected the marginal adaptation at the tooth–restoration interface in all experimental groups (Figure 7). Examination of the SEM images at 2000× magnification demonstrated that aging resulted in deterioration of surface characteristics in all restorative techniques, as evidenced by increased surface irregularities and morphological defects (Figure 8).

Among the evaluated techniques, the incremental technique and the incremental technique combined with brush application using a modeling liquid exhibited more favorable surface characteristics and improved marginal integrity compared with the other groups. In contrast, specimens restored using the bulk technique showed the most pronounced degradation in surface characteristics following aging.

## 4. Discussion

The present in vitro study evaluated the influence of four different placement techniques on microleakage, surface characteristics, wear behavior, and application time of a contemporary bulk-fill resin composite in standardized Class I cavities. The findings demonstrated that the placement technique significantly affected all evaluated parameters, leading to rejection of the null hypothesis.

Direct resin composite restorations account for a substantial proportion of routine clinical dental procedures [18]. Despite continuous material advancements and favorable outcomes reported in controlled clinical trials, the longevity of composite restorations remains highly variable in daily practice [19,20]. This variability is largely attributed to technique sensitivity, operator-related factors, and procedural complexity, which collectively influence marginal integrity, surface quality, and long-term clinical performance [21,22]. Consequently, optimizing placement techniques that balance efficiency with restoration quality remains a critical objective in restorative dentistry.

Conventional incremental layering has long been advocated as an effective strategy to reduce polymerization shrinkage stress, particularly in cavities with a high configuration factor. By decreasing the bonded surface area per increment and allowing stress dissipation during sequential polymerization, incremental techniques have been shown to improve marginal adaptation and reduce microleakage [23]. However, this approach is inherently time-consuming and technique-sensitive, increasing the risk of procedural errors such as void formation, interlayer contamination, and bonding inconsistencies [24]. These limitations have driven the development of simplified restorative approaches, including bulk-fill composites and anatomy-guided placement techniques.

In the present study, the bulk placement technique exhibited the shortest application time, consistent with its simplified clinical protocol that eliminates multiple layering and curing steps. This finding is in agreement with previous investigations reporting substantial reductions in operative time when bulk-fill approaches are employed. Notably, a previous laboratory study reported an approximately 49% decrease in application time for the bulk-fill base technique relative to incremental layering [25], while a recent randomized controlled clinical trial documented a 57% reduction in cavity-filling time when bulk-fill technique was employed [26]. Consistent with these reports, the findings of the present study revealed an almost 56% reduction in application time for bulk placement compared with the incremental technique.

Beyond bulk placement strategies, the stamp technique has gained attention as a biomimetic approach aimed at accurately reproducing occlusal anatomy while minimizing freehand sculpting. Precise restoration of cuspal morphology, fissures, and grooves is essential for maintaining occlusal stability and functional efficiency [27]. Freehand techniques may compromise these features and often necessitate extensive occlusal adjustment, which can adversely affect surface integrity and prolong chairside time. By transferring preoperative or diagnostically reconstructed occlusal morphology to the final composite increment under controlled pressure, the stamp technique facilitates anatomical accuracy, reduces the need for occlusal refinement, and limits surface defects associated with oxygen inhibition and porosity [28]. To the best of our knowledge, the present study is the first to quantitatively evaluate application time associated with the stamp technique. When compared with the conventional incremental technique, the stamp approach demonstrated an approximate 35% reduction in operative time, underscoring its potential to enhance procedural efficiency without increasing technical complexity. From a clinical standpoint, even relatively modest reductions in chairside time may confer meaningful advantages, including improved patient comfort, decreased operator fatigue, enhanced clinical workflow efficiency, and potential environmental benefits associated with reduced energy consumption [29]. Nevertheless, the time efficiency afforded by stamp and bulk placement techniques should be interpreted cautiously and in conjunction with their biological and mechanical consequences, as procedural simplification does not necessarily ensure optimal marginal integrity or long-term restorative performance.

Accordingly, to comprehensively assess the biological and mechanical consequences of the evaluated placement techniques, both microleakage scores and linear dye penetration measurements were performed using microCT. The results revealed that the bulk placement technique exhibited significantly greater microleakage compared with all other placement strategies. Although contemporary bulk-fill resin composites incorporate advanced resin matrices and stress-modulating monomer systems [30] and are widely reported to exhibit reduced polymerization shrinkage stress, the placement and anatomical contouring of a large composite volume in a single increment may impose inherent clinical and handling limitations. These challenges may compromise intimate adaptation of the material to the cavity walls during insertion, thereby increasing the likelihood of marginal debonding or gap formation. Consequently, suboptimal marginal adaptation represents a plausible mechanistic explanation for the elevated microleakage values observed in the bulk placement group. Collectively, these findings suggest that, in restorations characterized by a high configuration factor, such as Class I cavities, bulk placement may adversely affect marginal sealing performance, even when modern bulk-fill composite materials are employed.

In contrast, the incremental technique, incremental placement combined with modeling liquid, and the stamp technique demonstrated comparably low microleakage values. The favorable performance of incremental layering can be attributed to reduced shrinkage stress and improved adaptation associated with thinner composite layers [31]. Notably, the stamp technique achieved similar microleakage outcomes despite reduced procedure time compared with incremental methods. This finding may be explained by the controlled application of pressure during placement of the final increment, which likely enhances adaptation to cavity walls and minimizes interfacial voids [32]. Furthermore, the accurate reproduction of occlusal anatomy and preservation of harmonious occlusal contacts inherent to the stamp technique may contribute to a more favorable stress distribution under functional loading, thereby limiting stress concentration at the tooth–restoration interface. Collectively, these findings suggest that the stamp technique represents a clinically efficient placement strategy that preserves marginal integrity while offering meaningful procedural advantages over traditional incremental approaches.

The inclusion of a modeling liquid (GC Modeling Liquid) in one of the incremental groups warrants particular consideration. This material, composed primarily of UDMA- and HEMA-based monomers, was applied to facilitate handling and surface adaptation. While such agents are frequently used in clinical practice to improve sculptability and surface smoothness [33], their interaction with composite layers and potential effects on surface and interfacial properties remain incompletely understood. Available evidence suggests that modeling liquids may affect interlayer bond strength, monomer elution, water sorption, surface hardness, color stability, and surface characteristics in a manner highly dependent on their chemical composition and solvent content [34,35]. While several studies have reported adverse effects on color stability and microhardness following the application of modeling liquids, the overall body of literature addressing their physicochemical and mechanical implications remains limited [36,37]. Within this context, the findings of the present study are noteworthy, as the use of a modeling liquid did not result in increased microleakage compared with conventional incremental placement, suggesting that, when applied judiciously, its short-term use does not adversely compromise interfacial sealing.

In contrast to its neutral effect on marginal integrity, wear behavior differed significantly among the evaluated techniques. Specifically, the incremental technique combined with modeling liquid exhibited the highest wear values. This outcome may be attributed to alterations in surface composition and polymer network density associated with the formation of an unfilled or lightly filled resin-rich surface layer [38]. In addition, the relatively low filler content of this superficial layer may reduce resistance to mechanical wear. Furthermore, the presence of hydrophilic monomers may increase susceptibility to hydrolytic degradation, thereby accelerating surface loss under simulated wear conditions.

Conversely, the conventional incremental technique demonstrated the lowest wear values, which may reflect a more homogeneous filler distribution and optimal polymerization within each increment. The bulk and stamp techniques exhibited comparable wear behavior, suggesting that the use of a bulk-fill composite—when properly cured—does not inherently compromise wear resistance. Additionally, the reduced need for aggressive occlusal adjustment in the stamp technique may contribute to preservation of the original surface layer, which is known to influence wear performance.

Surface quality and marginal integrity remain clinically significant parameters, given their association with plaque accumulation, secondary caries, and restoration longevity. Although the precise threshold at which marginal discrepancies predispose to caries development remains uncertain, evidence suggests that adhesive systems may confine lesion progression to gap entrances, whereas larger defects are more likely to facilitate microbial colonization. Furthermore, the reduced need for extensive occlusal adjustment associated with the stamp technique may contribute to preservation of the original surface layer, a factor known to play a critical role in wear performance.

Several limitations of the present in vitro investigation should be acknowledged when interpreting the findings. First, although standardized Class I cavities were prepared to minimize variability, in vitro conditions cannot fully replicate the complex biological, thermal, chemical, and mechanical challenges of the oral environment. Factors such as pulpal pressure, saliva, biofilm formation, patient-specific occlusal dynamics, and intraoral temperature fluctuations may influence marginal integrity, wear behavior, and long-term restoration performance differently than laboratory simulations. Although cavity preparation followed a standardized 3 × 3 mm protocol verified using calibrated burs and periodontal probing, minor variations in cavity geometry may have occurred due to natural anatomical differences among molars and the technical challenges of maintaining perfectly defined internal geometry in deeper Class I preparations. Such subtle deviations are inherent to tooth-based experimental models and should be considered when interpreting the findings. Second, only a single bulk-fill composite material and a single adhesive system were evaluated; therefore, the results cannot be generalized to other bulk-fill formulations or bonding protocols with different resin chemistries, filler contents, or polymerization kinetics. Although combined mechanical loading and thermocycling were applied to simulate long-term clinical service, artificial aging protocols cannot entirely replicate the cumulative, multifactorial degradation processes that occur intraorally over extended periods. Moreover, the adhesive was applied strictly according to the manufacturer’s self-etch protocol without selective enamel etching. In clinical practice, selective phosphoric acid etching of enamel margins is frequently performed and may enhance enamel bond strength and marginal sealing. The absence of this adjunctive step may therefore limit the direct clinical extrapolation of the present findings.

Another limitation is that true volumetric three-dimensional leakage quantification could not be performed, as only standardized cross-sectional micro-CT image outputs were available for analysis. Consequently, leakage assessment was based on linear measurements obtained from the sections exhibiting the greatest dye penetration, which may not fully reflect the total extent of interfacial leakage. Furthermore, baseline micro-CT imaging was not conducted prior to aging. The micro-CT protocol required immersion of the specimens in silver diamine solution to enhance radiographic contrast and facilitate detection of interfacial discrepancies. This procedure irreversibly alters the specimens, rendering them unsuitable for subsequent mechanical loading and wear assessment. Because the experimental design required the same specimens to undergo aging and wear evaluation, micro-CT analysis was limited to the post-aging condition.

Future long-term clinical studies and investigations incorporating multiple materials, operators, and biological variables are therefore warranted to validate and extend the present findings.

## 5. Conclusions

Within the limitations of this in vitro study, the placement technique was shown to exert a significant influence on application time, microleakage behavior, surface characteristics, and wear performance of a contemporary bulk-fill resin composite restored in standardized Class I cavities. Although bulk placement provided the greatest procedural time efficiency, it was associated with significantly higher microleakage, suggesting compromised marginal adaptation in high C-factor cavities. In contrast, the incremental technique, incremental placement combined with modeling liquid, and the stamp technique demonstrated comparably low microleakage values, indicating more favorable marginal sealing. Among these, the stamp technique emerged as a clinically promising approach by achieving reduced application time without detriment to marginal integrity or wear resistance. The use of a modeling liquid did not adversely affect microleakage in the short term; however, it was associated with increased wear, underscoring the need for cautious clinical application. Overall, the findings highlight that procedural simplification alone does not guarantee optimal restorative performance and emphasize the importance of selecting placement techniques that balance clinical efficiency with long-term biological and mechanical reliability. Further long-term clinical investigations are required to corroborate these laboratory findings and to assess their relevance under intraoral conditions.

## Figures and Tables

**Table 1:** Restorative materials and equipment used in the present study.

Material	Type/Purpose	Manufacturer	Composition/Main Components
Filtek One Bulk Fill Restorative	Bulk-fill resin composite	3M, St. Paul, MN, USA	Fillers: Discrete silica nanoparticles (~20 nm); discrete zirconia nanoparticles (4–11 nm); zirconia–silica clustered fillers composed of 20 nm silica and 4–11 nm zirconia; ytterbium trifluoride agglomerates (~100 nm).Organic matrix: AFM with stress-relieving capability, AUDMA, UDMA, and 1,12-dodecane dimethacrylate
CLEARFIL SE BOND	Two-step self-etch adhesive	Kuraray Noritake Dental Inc., Tokyo, Japan	Primer: 10-MDP, HEMA, hydrophilic dimethacrylates, camphorquinone, amine accelerator, water.Bond: 10-MDP, Bis-GMA, HEMA, hydrophobic dimethacrylates, camphorquinone, amine accelerator, silanated colloidal silica
GC Modeling Liquid	Modeling liquid for composite manipulation	GC Corporation, Tokyo, Japan	Unfilled resin blend comprising UDMA, 2-hydroxyethyl methacrylate (HEMA), and 2-hydroxy-1,3-dimethacryloxypropane
Woodpecker iLed II	LED light-curing unit	Woodpecker Medical Instrument Co., Guilin, China	Light intensity: ~1000–1200 mW/cm^2^
Silver Nitrate (AgNO_3_)	Microleakage dye	Merck KGaA, Darmstadt, Germany	Aqueous silver nitrate solution (50%)
OraCheck Software v5.0.x	Wear analysis software	Dentsply Sirona, Bensheim, Germany	Digital surface comparison software
Micro-CT SkyScan 1272	Microleakage evaluation	Bruker MicroCT, Coventry, UK	High-resolution X-ray micro-computed tomography
SEM EVO LS10	Surface and marginal analysis	Carl Zeiss, Oberkochen, Germany	Scanning electron microscope

AUDMA: aromatic urethane dimethacrylate; AFM: addition–fragmentation monomer; UDMA: urethane dimethacrylate; 10-MDP: 10-methacryloyloxydecyl dihydrogen phosphate; HEMA: 2-hydroxyethyl methacrylate; SEM: scanning electron microscopy; Micro-CT: micro-computed tomography; LED: light-emitting diode.

**Table 2:** Comparison of application time, microleakage, and wear parameters according to composite placement technique (mean ± standard deviation).

Placement Technique	Application Time [s]	Microleakage Score	Linear Microleakage [mm]
Incremental technique	94.3 ± 9.9 ᵃ	0.75 ± 0.9 ᵃ	0.14 ± 0.2 ᵃ
Incremental technique + brush application with modeling liquid	87.6 ± 11.5 ᵃ	0.75 ± 0.8 ᵃ	0.13 ± 0.1 ᵃ
Bulk technique	41.8 ± 2.6 ᵇ	2.08 ± 0.5 ᵇ	0.28 ± 0.1 ᵇ
Stamp technique	61.7 ± 9.5 ᶜ	0.17 ± 0.4 ᵃ	0.03 ± 0.1 ᵃ
*p*-value	<0.001 ‡*	<0.001 †*	0.001 †*

‡ One-way analysis of variance (ANOVA), † Kruskal–Wallis test. *p* < 0.05. Different superscript letters within the same column indicate statistically significant differences (* *p* < 0.05).

**Table 3:** Comparison of ordinal microleakage scores according to placement techniques.

Placement Technique	Microleakage Scores	
	Median (Min–Max)	IQR
Incremental technique	0.5 (0–2) ᵃ	1.75
Incremental technique + brush application with modeling liquid	1 (0–2) ᵃ	1
Bulk technique	2 (1–3) ᵇ	0
Stamp technique	0 (0–1) ᵃ	0
*p*-value	<0.001 †*	

Values are presented as median (min–max) and interquartile range (IQR). †: Kruskal–Wallis test. * *p* < 0.05. Different superscript letters indicate statistically significant differences between groups.

## Data Availability

The original contributions presented in the study are included in the article, further inquiries can be directed to the corresponding author.

## Abbreviations

The following abbreviations are used in this manuscript:

CAD	Computer-aided design
AUDMA	Aromatic urethane dimethacrylate
AFM	Addition–fragmentation monomer
UDMA	Urethane dimethacrylate
10-MDP	10-methacryloyloxydecyl dihydrogen phosphate
HEMA	2-hydroxyethyl methacrylate
SEM	Scanning electron microscopy
Micro-CT	Micro-computed tomography
LED	Light-emitting diode
